# Effect of aromatase inhibitors on sex differentiation and embryonic development in chicks

**DOI:** 10.1002/vms3.623

**Published:** 2021-09-01

**Authors:** Salwan M. Abdulateef, Ahmad A. Majid, Mohammed A. Al‐Bayer, Srwd S. Shawkat, Ahmad Tatar, Thafer T. Mohammed, Firas M. Abdulateef, Mohammed Q. Al‐Ani

**Affiliations:** ^1^ Animal Production College of Agriculture University of Anbar Ramadi al‐Anbar Iraq; ^2^ Animal Sciences College of Agricultural Sciences University of Sulaimani Sulaimani Kurdistan Iraq; ^3^ Animal Science Research Department Golestan Agricultural and Natural Resources Research and Education Center, AREEO Gorgan Iran; ^4^ Ministry of Agriculture ‐ Directorate of Anbar Agriculture Ramadi Iraq; ^5^ Department of Biology College of Science University of Anbar Ramadi al‐Anbar Iraq

**Keywords:** aromatase, chicks, embryo, in ovo, sex differentiation

## Abstract

**Background:**

Sexual differentiation can occur after exposure to aromatase into the left gonad at 6.5 days of incubation. Aromatase inhibitors work by inhibiting the action of the aromatase, which converts androgens into estrogens by a process called aromatization.

**Objectives:**

The aim of this study was to investigate the effect of in ovo exposure to the aromatase inhibitor from tomato and garlic extract on sexual differentiation and embryonic development in chicken embryos.

**Methods:**

Three hundred eggs divided into five groups: Control 1 (CO; no injection); control 2 distilled water, DW; 0.1 ml/egg); garlic extract (GAR; 0.1 mg/egg); tomato extract (TOM; 0.1 mg/egg); and garlic and tomato extract mixed (ATM, 0.1 ml/egg). The solution was prepared and injected into the albumin from the thin end of the eggs on day five by using a 1 ml syringe with a 23‐gauge needle. The embryonic test (embryo/egg weight) conducted at 7, 14 and 17 days of incubation. After hatching, feather sexing conducted to determine the initial male. Chicks sex was later confirmed on day 42 by an optical microscope lens.

**Results:**

The results revealed that there was a significant increase (*p* < 0.01) in embryonic growth traits in all experimental treatments as compared to control treatments. There was a significant increase (*p* < 0.01) in the percentage of hatchability for all experimental treatments compared to control treatments and a significant increase (*p* < 0.01) in chick quality including one‐day‐old chick length and body weight. All experimental treatments showed a significant increase (*p* < 0.01) in the male‐to‐female ratio compared to control treatments.

**Conclusions:**

The effect of in ovo exposure to aromatase inhibitors stimulated female‐to‐male sex reversal and improved embryonic development.

## Graphical abstract

The aim of this study was to investigate the effect of in ovo exposure to the aromatase inhibitor of tomato and garlic extract on sexual differentiation and embryonic development of chicken embryos. Sexual differentiation can occur after exposure to aromatase into the left gonad at 6.5 days of incubation. Aromatase inhibitors work by inhibiting the action of the aromatase, which converts androgens into estrogens by a process called aromatization.

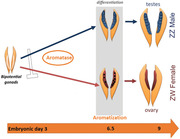

## 1 INTRODUCTION

In animals, sex is dependent on embryonic gonads differentiation and can be influenced by early exposure to sex steroid hormones (Correa et al., 2005). Birds differ from mammals in terms of how sex differentiation is genetically determined (Fazli et al., 2015). In birds, the female has a heterogametic sex chromosome ZW, while the male has a homogametic sex chromosome ZZ (Matsushita et al., 2006). The W chromosome controls early aromatase enzyme synthesis and leads to estrogen synthesis (Shimada, 1998, 2002). Estrogens and their receptors are important for sexual differentiation to determine female gender. Chicks’ embryonic gonads are bi‐potential at an early stage (Shimada, 2002). Like other bird species, female chickens will only develop the left gonad into a functional ovary (Romanoff, 1960). During female sexual development, the left gonad differentiates into a single ovary and oviduct, while the right is idle and regresses to generate the regular female phenotype (Lin et al., 1995). During sexual differentiation, testosterone will be converted to estrogen in response to aromatase enzyme expression in the left gonad at 6.5 days of incubation (Shimada, 1998; Yoshida et al., 1996). In male birds, both gonads will develop into a testis (Shimada, 1998). Sexual development relies on the presence of aromatase in steroid cell generation, which gauges the proportion of androgen hormones converted to estrogens through the gonads (Nishikimi et al., 2000). Low levels of P450 aromatase expression causes a shortage of estrogen synthesis in males (Nakabayashi et al., 1998; Shimada, 1998; Yoshida et al., 1996). The expression of the aromatase gene in females begins around day 5–6 of incubation, prompted by the synthesis of estrogen (Zhang et al., 1998). To create a functional left ovary, differentiation between right and left ovary in females especially depends on the estrogen receptor expression in the left gonad (Bruggeman et al., 2002). Sexual differentiation between female and male depends on the lack of aromatase and estrogen, while the estrogen receptor is available in males before sexual differentiation (Smith et al., 1997). Levels of steroid hormones are controlled by aromatase inhibition, which alters the last phase of sex steroid biosynthesis by changing androgens to estrogens (Elbrecht & Smith, 1992). Both male and female chicken embryos are capable of androgen synthesis, but only female embryos can create estrogen during the brief time period before the sexual differentiation of the gonads. Thus, the expression of P450 aromatase mRNA in the female chicken embryo has a critical role in the early phase of estrogen production. Studies on aromatase inhibitors included, for example, clomiphene and tamoxifen selective estrogen receptor modulators (Fazli et al., 2015), imazalil, and atrazine (Matsushita et al., 2006). The effects of these modulators have been tested on birds at different ages, starting at the early days of embryo growth (Robinzon et al., 1990) through 42 to 90 days of age (Rosenstrauch et al., 1986).

Tomato and its products contain high amounts of phytochemicals, which have health advantages and play an active role in nutrition due to antioxidant components. They also have natural aromatase inhibitors, for example lycopene, phenolics, flavonoids, phytoene, phytofluene, ascorbic acid, and the provitamin A‐carotenoid β‐carotene (Abushita et al., 1997; Rao & Agarwal, 1999). Lycopene is considered an acyclic unsaturated carotenoid due to its antioxidant properties and can be extracted from tomato (Takasova et al., 1995). In garlic, natural aromatase inhibitors are lignans and major phytoestrogen compounds (Valizadeh & Seratinouri, 2013). The natural compounds used in this study were intended to create sex differentiation in the chick embryo and to provide extra nutrients for the chick embryo to aid the hatching process.

The objective of the current study was to explore the impact of the natural extracts of tomato, garlic, and a combination of both, on sex differentiation, gonad growth, and embryonic development.

## 2 MATERIALS AND METHODS

### 2.1 Garlic extract

Garlic extract was obtained through steam distillation, for a short period, of mature garlic bulbs (*Allium sativum* L.) purchased from the Iraqi local market. The garlic bulbs were cleared of any adhering dried material, peeled, washed, and dried over drying paper, and thereafter chopped with a grinder. Then, 200 g of the prepared garlic was blended with 200 ml distilled water and put in a 1000 ml distillation flask attached to the steam distillation device. The steam distillation continued for 3 h at 100°C (Lee et al., 2003). The following chemical contents of garlic extract were determined: moisture, protein, lipid, ash, and fibre contents (AOAC, 2000). The carbohydrate content was estimated by adding the percentages of the aforementioned contents and subtracting the sum from 100. Ca, Fe, Zn, P, and K were determined by atomic absorption spectrophotometry (Mod AAnalyst 800; Perkin Elmer, CT, USA) using the corresponding standards. Vitamin C, pyridoxine (B6), glutamic acid, arginine, lysine, leucine, allicin, alliin, alkaloids, flavonoids, steroids, and cardenolides were determined as described (Guideline, 1994), which is shown in Table 1. Note that 0.1 ml of the hydrous garlic extract was injected into each treatment egg (Fazli et al., 2015). The in ovo injection is described below.

**TABLE 1:** Chemical composition (mg/L) of tomato and garlic extract (AOAC, 2000)

Garlic extract		Tomato extract	
Components	Value	Components	Value
Carbohydrate	73,220	Carbohydrate	60,290
Protein	15,330	Protein	12,220
Fibre	2100	Fibre	9250
Ash	4080	Ash	8110
Lipid	720	Lipid	3360
Moisture	4550	Moisture	6770
Vitamin C	31,100	Vitamin C	325,770
Pyridoxine (B6)	1,235,000	Riboflavin (B2)	617
Glutamic acid	850	Thiamin (B1)	639
Arginine	630	Pyridoxine (B6)	7390
Lysine	310	β‐Carotene	4990
Leucine	270	Lycopene	125,110
Alkaloids	4210	total phenolic compounds (TPC)	329,380
Allicin	9030	Calcium	18,990
Alliin	2,113,000	Zinc	1800
Flavonoids	5560	Potassium	2650
Steroids	40	Iron	850
Cardenolides	20		
Potassium	10,100		
Calcium	26,300		
Phosphorus	153,000		
Iron	5290		
Zinc	340		

### 2.2 Tomato material

The tomato extraction was prepared using an integrated SFE‐SFC system (10AVP; Shimadzu, Japan). A 25 g sample of crushed fresh tomato was placed into the extraction vessel, and a stream of carbon dioxide was passed through while the pressure and the temperature were maintained at 32 MPa and 50°C, as described previously (Gomez‐Prieto et al., 2002). The most insoluble and less volatile compounds were collected through the decrease in solvating power resulting from lowering the pressure (to 15 MPa) and increasing the temperature up to 80°C in a separation vessel (and consequently, from lowering the carbon dioxide density). The extract was recovered by washing the vessel with 5 ml dichloromethane. Extraction was repeated 10 times, and the total extracted material was diluted with 10 ml distilled water. Chemical composition of tomato extract includes the moisture, protein, lipid, ash, and crude fibre contents (AOAC, 2000). The carbohydrate content was estimated by adding the percentages of the aforementioned contents and subtracting the sum from 100. Ca, Fe, Zn, and K were determined by atomic absorption spectrophotometry (Mod Analyst 800; Perkin Elmer, CT, USA) using the corresponding standards. Vitamin C, riboflavin (B2), thiamin (B1), pyridoxine (B6), lycopene, and β‐carotene content as well as total phenolic content (TPC) were determined as described (Guideline, 1994), which is shown in Table 1. The extract was injected into the respective treatment eggs at 0.1 ml per egg (Fazli et al., 2015).

### 2.3 Tomato and garlic mixed extract

In the mixed treatment, 5 ml of garlic and tomato extracts were combined and blended in an electric mixer. The total amount was diluted with 10 ml distilled water, and 0.1 ml of the combined extract was injected into each treatment egg.

### 2.4 In ovo injection

Three hundred eggs were divided into five groups: control 1 (CO, no injection); control 2 distilled water (DW, 0.1 ml/egg); garlic extract (GAR, 0.1 mg/egg); tomato extract (TOM, 0.1 mg/egg); and garlic and tomato extract mixed (ATM, 0.1 ml/egg). Each treatment contained 60 eggs, 20 eggs per replicate. The materials were prepared and injected into the egg white (albumin) from the narrow end of the eggs on day 5 of incubation using a 1 ml syringe with a 23‐gauge needle. Injection sites on the eggs were cleaned with 70% ethyl alcohol, and then sealed by wax. In the control 2 group, 0.1 ml/egg of distilled water was injected into the eggs in a similar way. Eggs were incubated in Cimuka egg incubator (Turkey made) at 37.8°C and 65% humidity. Temperature decreased gradually to 36.5°C, while humidity increased to 85% in hatching day according to Ross‐308 management guide instructions. The eggs were automatically turned once per hour. The eggs were discarded if no developed embryo was detected by candling at day 10 of incubation.

### 2.5 Embryonic development tests, hatchability, and chick quality

The embryonic tests were conducted at day 7, 14, and 17 of incubation. Ten eggs from each replicate were taken for each assay and weighed in a sensitive electric balance (extra 30 eggs were added to each treatment for embryonic test and treated as same as eggs in original treatment). They were then broken in a petri dish and measured for the weight of the shell, embryo, albumen, yolk, membrane, and amniotic fluid. These weights were calculated as a percentage of the egg weight at the test, as described by (Orlov, 1987). The percentage of hatchability was determined as the number of chicks that hatched per number of fertile eggs in each set. After hatching, chicks of all treatment groups were measured for their body weight and length (Abdulateef, 2017).

### 2.6 Sexing analysis

After hatching, feather sexing was conducted as an initial test to the male and female percentage. Hatched 1‐day‐old chicks were reared up to 42 days of age, separated by sex, across 15 pens in a completely randomized design. There were five treatments, three replicates, and eight chicks per replicate. At the end of the experiment (day 42), three birds from each pen were slaughtered for carcass analysis. The gonads of the selected chicks were inspected by an optical microscope lens to confirm the sex and feather sexing conclusions on day 42 (Fazli et al., 2015).

### 2.7 Statistical analysis

In this experiment, 60 eggs were randomly assigned to each of the five investigated treatments, distributed across three replicates. Each of 20 eggs per replicate was under complete random design (CRD). Data were analyzed using a general linear model (SAS, 2012), with injection treatments as a fixed effect. Means were compared according to Duncan's polynomial using different significance levels to determine significant differences between treatment means (D. B. Duncan, 1955) development of broiler chickens.

## 3 RESULTS

### 3.1 Embryonic development

Table 2 shows the effect of in ovo exposure to aromatase inhibitors at day 7 of incubation on embryonic development. ATM showed a significant increase (*p* < 0.01) in embryonic weight (2.26%) compared to the other treatments, while GAR (1.76%) and TOM (1.77%) treated eggs had a significant increase (*p* < 0.01) in embryonic weight compared to CO eggs. There was no significant difference between DW eggs and any other group and also in the combined amniotic weight+fluid between the experimental groups and control groups. However, there was a significant increase (*p* < 0.01) in allantoic weight+fluid between GAR, TOM, and ATM eggs 6.74, 7.84, and 6.87%, respectively, compared to CO and DW eggs. ATM eggs had a significant decrease (*p* < 0.01) in albumin weight (8.50%) and shell weight (7.56%) compared to the other treatments. There was no difference between the experimental groups and control groups in yolk weight.

**TABLE 2:** The effect of in ovo exposure to the aromatase inhibitors on development at 7 days from incubation

As a percentage of the egg weight at the test (%)								
	Treatments							
Traits (gm)	CO	DW	GAR	TOM	ATM	SEM	Mean	*p*‐Value
Embryonic weight	1.13^c^	1.37^bc^	1.67^b^	1.77^b^	2.62^a^	0.222	1.71	*
Amniotic weight+fluid	3.35	3.84	3.43	3.55	3.45	0.238	3.52	N.S.
Allantoic weight+fluid	4.58^b^	4.56^b^	6.74^a^	6.84^a^	6.87^a^	0.637	5.92	^*^
Albumin weight	10.79^a^	10.29^ab^	9.38^bc^	10.09^ab^	8.50^c^	0.619	9.81	*
Yolk weight	16.89	17.63	18.00	17.45	17.33	1.11	17.46	N.S.
Shell weight	9.48^a^	9.95^a^	9.27^a^	8.72^ab^	7.65^b^	0.667	9.01	^*^

*Note*: The different superscript letters in the same rows differ significantly at probability values 0.01 and 0.05.

Control 1: no injection (CO); control 2: distilled water (DW, 0.1 ml/ egg); garlic extract (GAR, 0.1 mg/ egg); tomato extract (TOM, 0.1 mg/ egg); and garlic and tomato mixed extract (ATM, 0.1 ml/ egg).

Abbreviations: N.S., non‐significant; SEM, standard error mean.

* *p* < 0.01.

Table 3 presents the effect of in ovo exposure to aromatase inhibitors at incubation day 14 on embryonic development. ATM eggs had a significant increase (*p* < 0.01) in embryonic weight (19.46%) compared to GAR (18.06%) and TOM (17.53%) eggs, which was higher than CO and DW. There was a significant increase (*p* < 0.01) in amniotic weight+fluid in ATM eggs (14.20%) compared to other treatments except for TOM. The allantoic weight+fluid significantly increased (*p* < 0.01) in GAR (11.83%), TOM (12.33%), and ATM (13.16%) eggs compared to CO and DW eggs. There was also a significant decrease (*p* < 0.01) in albumin weight in GAR (5.60%), TOM (5.23%), and ATM (5.03%) eggs compared to CO and DW eggs. There was a significant decrease (*p* < 0.01) in yolk weight in ATM eggs (12.16%) compared to the other treatments, except for GAR and TOM eggs. The shell weight significantly decreased (*p* < 0.01) in ATM eggs (6.35%) compared to the other treatments.

**TABLE 3:** The effect of in ovo exposure to the aromatase inhibitors on embryonic development at 14 days from incubation

	Treatments							
Traits(gm)	CO	DW	GAR	TOM	ATM	SEM	Mean	*p*‐Value
Embryonic weight	13.30^c^	13.14^c^	18.06^b^	17.53^b^	19.46^a^	0.741	16.30	*
Amniotic weight	12.70^b^	13.03^b^	10.33^c^	13.33^ab^	14.90^a^	0.901	12.86	*
Allantois weight	8.33^b^	8.80^b^	11.83^a^	12.33^a^	13.16^a^	1.02	10.89	*
Albumin weight	7.33^a^	7.60^a^	5.60^b^	5.23^b^	5.03^b^	0.846	6.16	*
Yolk weight	15.00^a^	14.10^ab^	13.00^bc^	13.66^abc^	12.16^c^	0.844	13.58	*
Shell weight	8.35^a^	8.42^a^	8.06^a^	7.72^a^	6.53^b^	0.423	7.81	*

*Note*: The different superscript letters in the same rows differ significantly at probability values 0.01 and 0.05.

Control 1: no injection (CO); control 2: distilled water (DW, 0.1 ml/ egg); garlic extract (GAR, 0.1 mg/ egg); tomato extract (TOM, 0.1 mg/ egg); and garlic and tomato mixed extract (ATM, 0.1 ml/ egg).Abbreviation: SEM, standard error mean.

* *p* < 0.01.

Table 4 shows the effect of in ovo exposure to aromatase inhibitors at day 17 of incubation on embryonic development. ATM eggs had a significant increase (*p* < 0.01) in embryonic weight (29.67%) compared to the other treatments. There was a significant increase (*p* < 0.01) in amniotic weight+fluid in TOM (17.53%) and ATM (18.16%) eggs compared to the other treatments except for GAR eggs. The allantoic weight+fluid significantly increased (*p* < 0.01) in GAR (25.15%), TOM (24.85%), and ATM (26.18%) eggs compared to CO and DW eggs. Yolk weight significantly decreased (*p* < 0.01) in TOM (13.18%) and ATM (11.96%) eggs compared to CO and DW eggs, while GAR (15.13%) eggs had a significantly lower yolk weight than DW eggs. The mixed garlic and tomato extract treatment (ATM) significantly decreased (*p* < 0.01) shell weight (6.21%) compared to the other treatments.

**TABLE 4:** The effect of in ovo exposure to the aromatase inhibitors on embryonic development at days 17 of incubation

	Treatments							
Traits(gm)	CO	DW	GAR	TOM	ATM	SEM	Mean	*P*‐value
Embryonic weight	24.00^d^	25.86^c^	27.26^b^	27.56^b^	29.67^a^	0.520	26.87	*
Amniotic weight	15.66^c^	16.00^bc^	17.16^ab^	17.53^a^	18.16^a^	0.688	16.90	*
Allantois weight	21.04^b^	21.87^b^	25.15^a^	24.85^a^	26.18	0.804	23.82	*
Yolk weight	16.00^ab^	17.20^a^	15.13^bc^	13.80^c^	11.96^d^	0.906	14.82	*
Shell weight	7.60^a^	7.57^a^	7.17^a^	7.43^a^	6.21^b^	0.246	7.19	*

*Note*: The different superscript letters in the same rows differ significantly at probability values 0.01 and 0.05.

Control 1: no injection (CO); control 2: distilled water (DW, 0.1 ml/ egg); garlic extract (GAR, 0.1 mg/ egg); tomato extract (TOM, 0.1 mg/ egg); and garlic and tomato mixed extract (ATM, 0.1 ml/ egg). Abbreviation: SEM, standard error mean.

* *p* < 0.01.

### 3.2 Hatchability and chicks quality

Table 5 shows the effect of in ovo exposure to aromatase inhibitors on hatchability and chick quality. The results show a significant increase (*p* < 0.01) in the percentage of hatchability in ATM eggs (77.00%) compared to the other treatments. Chicks hatched from the ATM treatment also had significantly higher (*p* < 0.01) body length (20.36 cm) and weight (40.73 mg) compared to the other treatments.

**TABLE 5:** The effect of in ovo exposure to aromatase inhibitors on the hatchability and chick's quality

	Treatments							
Traits	CO	DW	GAR	TOM	ATM	SEM	Mean	*P*‐value
Hatchability (%)	66.66^d^	67.66^cd^	69.00^c^	72.00^b^	77.00^a^	1.15	70.46	*
Length (cm)	17.30^c^	17.66^c^	19.26^b^	18.90^b^	20.36^a^	0.589	18.69	*
Weight (gm)	34.73^d^	35.30^cd^	38.23^b^	37.76^b^	40.73^a^	0.755	37.35	*

*Note*: The different superscript letters in the same rows differ significantly at probability values 0.01 and 0.05.

a, b, c, d: means in the same rows with different superscripts differ significantly at probability value p≥ 0.01 and 0.05.

Control 1: no injection (CO); control 2: distilled water (DW, 0.1 ml/ egg); garlic extract (GAR, 0.1 mg/ egg); tomato extract (TOM, 0.1 mg/ egg); and garlic and tomato mixed extract (ATM, 0.1 ml/ egg).

Abbreviation: SEM, standard error mean.

* *p *< 0.01.

### 3.3 Sexing analysis

**FIGURE 1:**
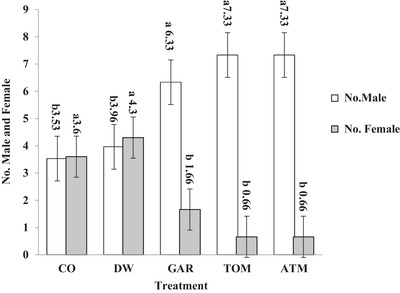
The effect of in ovo exposure to aromatase inhibitors on the number of male and female of broiler chickens. *SEM: 0.730 to male and 0.730 to female. *Mean: 5.69 to male and 2.17 to female. a and b: mean in the same rows with different superscripts differ significantly at probability value 0.01 and 0.05. *Control 1: no injection (CO); control 2: distilled water (DW, 0.1 ml/egg); garlic extract (GAR, 0.1 mg/egg); tomato extract (TOM, 0.1 mg/egg); and garlic and tomato mixed extract (ATM, 0.1 ml/egg). *Injected into the eggs in 5‐day incubation

**FIGURE 2:**
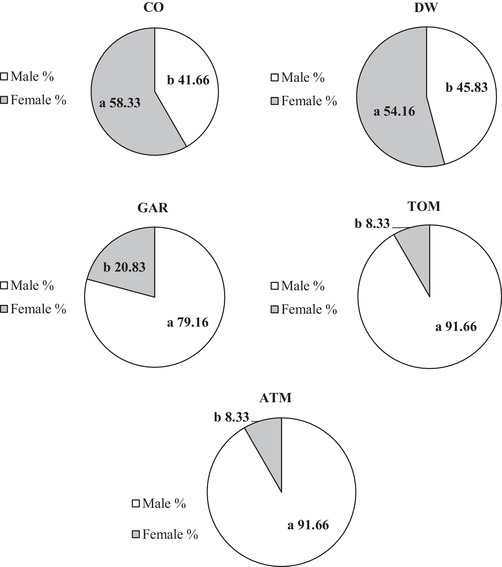
The effect of in ovo exposure to aromatase inhibitors on the percentage of female to male of broiler chickens. † SEM: 9.12 to male and 9.12 to female. ‡ Mean: 70.0 to male and 30.0 to female. a and b: mean in the same rows with different superscripts differ significantly at probability value 0.01 and 0.05. *Control 1: no injection (CO); control 2: distilled water (DW, 0.1 ml/egg); garlic extract (GAR, 0.1 mg/egg); tomato extract (TOM, 0.1 mg/egg); and garlic and tomato mixed extract (ATM, 0.1 ml/egg). * Injected into the eggs in 5‐day incubation

Figure 1 shows the effect of in ovo exposure to aromatase inhibitors on the number of male and female broiler chickens. The number of males was significantly higher (*p* < 0.01) in GAR, TOM, and ATM treatments compared with CO and DW treatments. Figure 2 shows the effect of in ovo exposure to aromatase inhibitors on the percentage of female and male broiler chickens, with a greater percentage of males (*p* < 0.01) in GAR, TOM, and ATM treatments compared to CO and DW treatments.

## 4 DISCUSSION

### 4.1 Embryonic development

The bioactive materials of garlic (*A. sativum* L.) are allicin, allinase, thiosulphonate, 1‐propenyl allyl thiosulphonate, and γ‐l‐glutamyl‐S‐alkyl‐l‐cysteine. These effective compounds influence feed intake, feed utilization, body weight, and blood lipid profiles. Enzymes present in garlic are activated and act upon alliin to create allicin thereby affecting embryonic growth. Moreover, there are essential sulphur‐containing materials existent in garlic (allyl methyl). These regulate cholesterol levels in chickens energize the immune system, promote detoxification of exotic compounds, and have anti‐inflammatory and antioxidant effects (Puvača et al., 2015). Allicin has been shown to decrease low‐density lipoprotein, cholesterol, and triglyceride in serum (Alder & Holub, 1997) and body tissues (Stanaćev et al., 2012). It also has a role in preventing bacterial growth (Griffin et al., 1992) and moderating oxidative stress (Ide & Lau, 2001). In broilers, adding garlic as a feed supplement improved their growth and feed conversion ratio (FCR) and reduced the mortality rate (Stanaćev et al., 2010). Garlic contains the minerals Ca, Fe, K, Cu, Mg, and a variety of vitamins such as thiamine (B1), riboflavin (B2), niacin (B3), pantothenic acid (B5), vitamin B6, folate (B9), and vitamin C as well as carbohydrates, sugars, fat, protein, and amino acids (Shojai et al., 2016). These contents contribute to embryo development, improve feeding, and reduce mortality (Tollba & Hassan, 2003). Garlic has also been used as an antibiotic growth promoter in chickens (Dibner & Richards, 2005), while Raeesi et al. (2010) used 1–3 g/kg garlic powder diets to improve growth performance. Choi et al. (2010), however, found no effect on growth performance when adding garlic, contrasting with the studies of Pourali et al. (2010) and Varmaghany et al. (2015) who found a positive effect of garlic on chick production performance (Shojai et al., 2016). In line with the previous positive results (Suleria et al., 2015), in this study we indeed found increased embryo weight in the garlic treatment (GAR) and the treatment group with tomato and garlic mixed extract (ATM).

The success of embryonic development is determined by the composition of the hatching egg, early feeding, and the immune status. The process of hatching is stressful to the embryo and can be affected by other stressors, especially heat stress. Birds exposed to heat stress increased their lipid peroxidation and depress their growth, while there was an increase in free radicals (Fisher & Kemp, 2000). Injecting lycopene, which is found in tomato, into the egg causes free radical inhibitor activity, creating protection against lipid peroxidation in the egg yolk (Jiang et al., 2015). This agrees with the study of Takasova et al. (1995) which shows that the tomato contains fatty acids including palmitic acid (16:0), stearic acid (18:0), and oleic acid (18:1n‐9). Tomatoes are also considered a source of vitamins and may maintain vitamin levels due to antioxidant impact (Jiang et al., 2015).

Lycopene also contributes to egg stability and the content of the egg yolk, helping the embryo grow (N. Sahin et al., 2006). It has the strongest antioxidant activity among carotenoids due to its high number of conjugated double bonds (Jiang et al., 2015). Lycopene can give protection against cell harm caused by reactive oxygen species (ROS) (Jain et al., 1999). Therefore, it can be given to prevent cell and tissue damage and additionally improve hereditary problems (Prakash & Kumar, 2013). Consuming lycopene has a cardio‐protective effect on humans and animals (Rissanen, 2006) by up‐controlling oxidation status. For example, it enhances the activity of antioxidant enzymes and antioxidant vitamin contents (Luo & Wu, 2011) and the plasma lipid profile (Upaganlawar & Balaraman, 2012). Jiang et al. (2015) showed that lycopene antioxidants protect against the influence of enzymes such as superoxide dismutase (SOD), glutathione peroxidase (GSH‐Px), and catalase (CAT) and non‐enzymes such as vitamins A and E. Lycopene plays an important role in inactivating hydrogen peroxide and nitrogen dioxide (Böhm et al., 1995). Furthermore, tomato pomace containing 1.3% lycopene was able to offset oxidative stress in animals by increasing transcription of genes that contribute to oxidative resistance (K. Sahin et al., 2014). Boileau et al. (2002) mentioned that lycopene is better absorbed from the intestinal wall due to rapid association with the bile that forms the emulsifier (Ahuja et al., 2006). This explains the increased absorption of lycopene by eating high‐fat diets through stimulating the production of bile (Skiepko et al., 2016). Using tomatoes in nutrition led to improved performance because lycopene increases protein synthesis and proteolysis in the broiler muscle (Hayashi et al., 1994). Surai et al. (1996) showed that lycopene moves from the yolk to embryotic tissues a few days before hatching, increasing lipid unsaturation and protecting the embryo against lipid peroxidation. Increased feed utilization, as well as increased lycopene, will increase coenzymes levels and could positively affect immunocompetence in the developing chick (Karadas et al., 2006; Surai et al., 2016). Injecting the egg with tomato extract would give the embryo better protection against oxidation, improved embryonic tissue, and increased growth and development (Gao et al., 2016). In line with the above, we found increased weight in embryos injected with tomato extract (TOM) or a combination of tomato and garlic extract (ATM).

In ovo injection of Chinese painted quail (*Coturnix Chinensis*) with 60 ng of testosterone induced embryo growth and reduced hatchling immune levels (Anderson et al., 2004). High testosterone concentration in house finch eggs stimulated bone growth factors and cartilage cells in chicks (Navara et al., 2006).

### 4.2 Hatchability and chicks quality

Hatchability depends on the quality of the embryo during the hatching period. Improving embryo quality requires less energy depletion and can lead to better chick welfare (Abdulateef, 2017). The results showed an improvement in embryo quality due to lycopene and garlic, resulting in improved hatchability. The qualities of garlic that were described above will aid the embryo in overcoming the stress of hatching (Shojai et al., 2016). Lycopene is thereby beneficial to promote nutrient absorption in the intestine due to villus length (Koutsos et al., 2003). The aforementioned qualities of lycopene improve vital processes in the embryo thus improving the hatchability (Sun et al., 2015). Chick length is one method to evaluate chick quality. It is a valid predictor of chick development because it is positively linked to yolk‐free body mass at hatching. Longer chicks were also heavier, which can be explained by the relationship between weight and length in poultry (Petek et al., 2008). Embryo activity during the hatching period will promote increased body weight and length (Farner et al., 2012). Egg yolk hormone levels may affect sex ratio of hatching chicks by reducing embryo mortality in one gender without affecting the other (Li et al., 2008; Navara, 2013). Rubolini et al. (2006) showed that in ovo injection of yellow leg gulls with testosterone hormone caused high female embryo mortality thus increasing hatching males.

### 4.3 Sexing analysis

The reproductive system of male birds (semen production and testes activity) is affected by several factors such as age, season, nutrition, photoperiod, daily rhythm, management system, genetics, and health (Edens, 2011). There are two hypotheses regarding sex development in birds. The first is the ‘Z dosage’ hypothesis and is based on the perception that avian species have no dosage compensation system for the Z chromosome (Itoh et al., 2010). Thus, it is suggested that high levels of Z‐linked genes in the gonads of ZZ embryo result in testis development. There is a stable gene for sex development, which is the Z‐linked double sex and mab‐3‐related transcription factor 1 (DMRT1) gene. The second is the ‘W dominant’ hypothesis in which the W chromosome contains a dominant‐acting ovary determinant or an inhibitor of testis differentiation. histidine triad nucleotide‐binding protein W (HINTW) was the most influential ovary determining gene W‐linked ovary‐determining gene also known as WPKCI and ASW (Sasanami, 2017).

The natural aromatase inhibitors in tomato, such as lycopene and flavonoids, and in garlic, such as lignans and major phytoestrogen compounds, are interesting examples that illustrate their mode of action. These results agree with Smith et al. (1997) who showed that sex differentiation between female and male depends on the lack of aromatase and estrogen, while the estrogen receptor is available in males before sexual differentiation. Both endocrine and growth factor pathways modify steroid hormone metabolism, which modifies insulin‐like growth factors and estimated glomerular filtration rate (Dabrosin et al., 2002) to inhibit aromatase and 17β‐hydroxysteroid dehydrogenase. Lignans inhibit cell proliferation in both estrogen receptor positive and negative cell lines and work synergistically with flavonoids to reduce estrogen receptor growth (Wang et al., 1994). Decreased estrogen prevents left ovarian duct development and right duct regression (Brooks & Thompson, 2005). The expression of aromatase gene in females begins at about 5–6 days of incubation, prompted by the synthesis of estrogen (Zhang et al., 1998) and particularly the estrogen receptor mRNA in the left gonad (Nakabayashi et al., 1998), which explains the results in this study. On the other hand, aromatase in the brain is usually only expressed in neurons, which is thought to be due to the neuroprotective actions of estrogens, including estradiol. Research has found that two cytokines, interleukin‐1β (IL‐1β) and interleukin‐6 (IL‐6), are responsible for aromatase expression in astrocytes following brain penetration in the zebra finch (K. A. Duncan & Saldanha, 2011). Therefore, lycopene could be considered an aromatase inhibitor by reducing interleukin production in the body and preventing aromatase formation (Gouranton et al., 2011). Lycopene has 5‐alpha‐reductase inhibiting properties, and therefore works similarly to well‐known chemical inhibiting drugs (Gouranton et al., 2011). Lycopene interferes with local testosterone receptor activation by down‐regulating 5‐α‐reductase and consequently reduces steroid target gene expression (cystatin‐related protein 1 and 2, prostatic spermine binding protein, prostatic steroid binding protein C1, C2, and C3 chain, and probasin) (Jiang et al., 2015). Flavonoids work as an estrogen antagonist and bind to the estrogen receptors in cells, blocking estrogen in the body ultimately inhibiting growth. Because of this, flavonoids are considered selective estrogen receptor modulators (Pelissero et al., 1996).

Garlic is a dietary source of lignans which contain aromatase inhibitors (Valizadeh & Seratinouri, 2013). It has been suggested that lignans inhibit estrogen synthesis and compete with estradiol for the estrogen receptor and type II binding sites (Ibrahim & Abul‐Hajj, 1990). Lignans contain enterolactone (Enl) and its precursors 3′‐demethoxy‐3O‐demethylmatairesinol (DMDM) and didemethoxymatairesinol (DDMM), which decrease aromatase enzyme activity. Its precursors contain a keto‐tetrahydrofuran ring. The ring structure may either directly increase the Enl's affinity to the aromatase enzyme, or it may increase lipid solubility, allowing Enl and its theoretical precursors to enter the cell more easily. Once in the cell, Enl can access the binding site of aromatase and block its pathway (Wang et al., 1994). The P450 aromatase involved in mRNA expression in the female gonad begins to form in the ovary at 6–7 days of incubation, but there is no such expression in the male chick gonads (Akazome et al., 2002; Vaillant et al., 2001; Yamamoto et al., 2003). Cunningham and Russell (2001) showed that hormones are transferred from mother peahens to their eggs at various levels. Eggs containing male embryos had significantly higher androgen levels, and eggs containing female embryos had significantly higher levels of 17‐β estradiol. In male chicks, anti‐Mullerian hormone (AMH) might be involved in higher mRNA expression in the gonad and initiate the testes formation, whereas in females it has a lower expression (Nishikimi et al., 2000; Oréal et al., 2002). Testes development and sex differentiation in males are therefore more dependent on AMH and androgen secretion from testes (Shimada, 1998). AMH is secreted from Sertoli cells and prohibits the development of the Müllerian duct and thus the reproductive system of females (Wibbels et al., 1992). The levels of steroid hormones are controlled by aromatase inhibition, which marks the last phase of sex steroid biosynthesis by changing androgens to estrogens (Elbrecht & Smith, 1992). Both male and female chicken embryos are able to synthesize androgens, while female embryos can create estrogen during the brief time frame before sexual differentiation of the gonads. Thus, the expression of P450 aromatase mRNA in the female chicken embryo has a critical role in the early phase of estrogen production. Studies in rodents demonstrated that testosterone has a sex specific influence on the brain with respect to aromatization, and controlling androgen sensitivity, within brain regions that mediate male sexual behaviour. Al‐Bayar (2016) showed that injecting Iraqi native hens with 500 µg testosterone per week produced significantly more male chicks than female chicks. Studies have been carried out on various antiestrogenic compounds used for the control of physiological processes (Roselli & Klosterman, 1998).

Phytoestrogens are a diverse group of non‐steroidal compounds that occur naturally in many plants. Because they possess a ring system similar to estrogens, they are able to bind to estrogen receptors (Edmunds et al., 2005). The sexual differentiation happens because of aromatase expression and generation of estrogen from the testosterone in the left gonad at 6.5 days (Shimada, 1998; Yoshida et al., 1996). In the right gonads in both male and female, however, the cortex is not existent by 6 days of incubation due to the absence of estrogen receptor gene expression (Fazli et al., 2015). On the other hand, the gene transcript for aromatase is present in the regressing right gonad (Nakabayashi et al., 1998). Consequently, aromatase has an effective role in the regression of the medulla of the right gonad in birds, and it changes testosterone to estradiol (Shimada, 1998). Exposing the chick embryo to aromatase inhibitors has been shown to defeminize the ovary and accessory structures (Fazli et al., 2015; Matsushita et al., 2006; Ottinger & Vom Saal, 2002). Female chickens that developed testes were capable of spermatogenesis and possessed the physical appearance and behaviour of males (Elbrecht & Smith, 1992). The aromatase protein is expressed in the medulla of female gonads from 6.5 days onwards and its expression increases during ovarian development (Smith et al. 1999, 2005). In the last part of estrogen biosynthesis specifically, androstenedione is converted to estrone and testosterone to estradiol through three progressive hydroxylations of the 19‐methyl group of androgens, with synchronous evacuation of the methyl group as format and aromatization of the A‐ring (Elbrecht & Smith, 1992). The activity of aromatase depends on factors including age, insulin, obesity, and alcohol. Aromatase movement is diminished by prolactin, the AMH, and the herbicide glyphosate. Aromatase activity is shown to be high in certain estrogen‐dependent local tissue (Gasnier et al., 2009). Aromatase is additionally oversensitive to environmental impacts, especially temperature. In species which depend on temperature for sex determination, aromatase expression is higher at temperatures that yield females (Duffy et al., 2010). Tomato and its products contain phytochemicals, which have health advantages and include antioxidant components such as lycopene, phenolics, flavonoids, phytoene, phytofluene ascorbic acid, and the provitamin A‐carotenoid β‐carotene (Abushita et al., 1997; Rao & Agarwal, 1999). Vitamin A is necessary for the synthesis of retinoic acid (RA). RA is a metabolite of vitamin A (retinol) that mediates the functions of vitamin A required for growth and development and is key to controlling meiotic initiation in many animal species. RA is produced in animal embryos through the tissue‐specific expression of three enzymes of retinaldehyde dehydrogenase (RALDH), namely RALDH1, RALDH2, and RALDH3. In chick embryos, RALDH2 is the main enzyme in charge of RA synthesis, and sites of RALDH2 gene expression are associated with RA production (Blentic et al., 2003). However, RALDH2 is firmly expressed in the gonads of both sexes from 6.5 days of incubation (phase 30). In male chicks, the expression of RALDH2 restricts the seminiferous ropes, whereas in females it limits the cortex production in the left gonad and the medulla of the right gonad (Smith et al. 2008). RA led meiotic entry in developing chicken gonads through the expression of RADH2, a major RA synthesizing enzyme, and cytochrome P450 family 26, subfamily B member 1, which is a major retinoic acid‐degrading enzyme (Sasanami, 2017).

Initial expressions of DMRT1 and SOX9 occur at different times in birds between 3.5 and 6.5 days, respectively. SOX9 regulates transcription of the AMH gene (Oréal et al., 2002). SOX9 also plays an important role in male sexual development through steroidogenic factor 1 (SF‐1) protein, a transcription factor involved in sex determination by controlling the activity of genes related to the reproductive glands, gonads, or adrenal glands (Parker & Schimmer, 1997). SOX9 produces AMH in the Sertoli cells to prevent the female reproductive system from developing. It is also associated with other genes that enhance the sexual organs of males (Sekido & Lovell‐Badge, 2008). This commences when the testis‐determining factor encoded by the sex‐determining region SRY of the Y chromosome activates SOX9 (Moniot et al., 2009).

AMH is a glycoprotein pertinent to the transforming growth factor‐β (TGF‐β) superfamily. It is secreted by the gonads and plays a vital part in sexual differentiation of reproductive organs. It is synthesized and secreted by the Sertoli cells of the embryonic testis and regresses the paired Müllerian ducts of males (Josso & Picard, 1986). In mammals, SOX9 directly regulates the AMH transcription together with NR5A1, GATA4 (GATA binding protein 4), and WT1 (Wilms tumour protein homolog). However, in chickens, AMH mRNA expression occurs prior to that of SOX9. AMH mRNA likewise exists in the female gonads of embryonic chickens and the right female Müllerian ducts (Hutson et al., 1981). It is thought that estrogens shield the left duct from regression through AMH (Josso et al., 2001).

Sexual differentiation happens in chickens and many other bird species. When female birds are masculinized, sex reversal occurs in ZW females between 0 and 7.5 days. During this time, gonads are developing, and a testis‐like structure with a thick cortex and bushy medulla can be observed despite the fact that the embryo is genetically female (ZW) (Kuroiwa, 2018). Genes involved in testis differentiation are unregulated, whereas female marker genes are down‐regulated. There have been no cases reported of male‐to‐female sex inversion in avian species under natural conditions, showing that the avian cannot be female without the W chromosome. This conclusion strongly supports the W dominant hypothesis, which preserves that W‐linked genes dominantly determine ovary differentiation (or inhibit testis differentiation).

In conclusion, injecting garlic and tomato extracts into eggs before hatching resulted in a skewed sex ratio in the chicken. We therefore conclude that garlic extract, tomato extract, and the combination of both showed an aromatase inhibitor effect in the embryonic development. The extracts are in addition a source of nutrients for chicks. They can be used in hatcheries as natural compounds to increase the male‐to‐female ratio in broiler chicks.

## ETHICS STATEMENT

The authors confirm that the ethical policies of the journal, as noted on the journal’s author guidelines page, have been adhered to and the appropriate ethical review committee approval has been received. The US National Research Council's guidelines for the Care and Use of Laboratory Animals were followed.

## AUTHOR CONTRIBUTIONS

S. M. Abdulateef designed the conceptualization, as well as participated in the investigation, writing–original draft, writing–review & editing, project administration–management and supervision. A. A. Majid participated in data curation and investigation. M. A. Al‐Bayer participated in writing–original draft and visualization–preparation. S. S. Shawkat participated in resources–provision of study materials and project administration. A. Tatar participated in writing–review & editing. Th. T. Mohammed participated in software–programming, software development, and formal analysis–application of statistical. F. M. Abdulateef participated in data curation–management activities to annotate (produce metadata). M. Q. Al‐Ani participated in validation–verification, whether as a part of the activity or separate, of the overall replication/reproducibility of results/experiments and other research outputs.

### PEER REVIEW

The peer review history for this article is available at https://publons.com/publon/10.1002/vms3.623

## Data Availability

The data that support the findings of this study are available in the supplementary material of this article.

## References

[vms3623-bib-0001] Abdulateef, S. M. (2017). The effect of maternal care (acoustic stimulus) on embryonic development and hatching muscle of broiler chickens. Iraqi Journal of Agricultural Sciences, 48(5), 1263–1274. https://jcoagri.uobaghdad.edu.iq/index.php/intro/article/view/336

[vms3623-bib-0002] Abushita, A. A. , Hebshi, E. A. , Daood, H. G. , & Biacs, P. A. (1997). Determination of antioxidant vitamins in tomatoes. Food Chemistry, 60(2), 207–212.

[vms3623-bib-0003] Adler, A. J. , & Holub, B. J. (1997). Effect of garlic and fish‐oil supplementation on serum lipid and lipoprotein concentrations in hypercholesterolemic men. The American Journal of Clinical Nutrition, 65(2), 445–450. 9022529 10.1093/ajcn/65.2.445

[vms3623-bib-0004] Ahuja, K. D. , Pittaway, J. K. , & Ball, M. J. (2006). Effects of olive oil and tomato lycopene combination on serum lycopene, lipid profile, and lipid oxidation. Nutrition, 22(3), 259–265. 16413753 10.1016/j.nut.2005.07.015

[vms3623-bib-0005] Akazome, Y. , Abe, T. , & Mori, T. (2002). Differentiation of chicken gonad as an endocrine organ: Expression of LH receptor, FSH receptor, cytochrome P450c17 and aromatase genes. Reproduction, 123(5), 721–728. 12006100

[vms3623-bib-0006] AL‐Bayar, M. A. (2016). Effect of testosterone injection to native layer breeder Mezo on primary and secondary sex ratio, fertility and hatchability. Anbar Journal of Agricultural Sciences, 14(1), 94–102. www.ajas.uoanabr.edu.iq

[vms3623-bib-0007] Andersson, S. , Uller, T. , Lõhmus, M. , & Sundström, F. (2004). Effects of egg yolk testosterone on growth and immunity in a precocial bird. Journal of Evolutionary Biology, 17(3), 501–505. 15149393 10.1111/j.1420-9101.2004.00706.x

[vms3623-bib-0008] AOAC . (2000). Official methods of analysis of the association of official analytical chemists (17th ed.). Association of Official Analytical Chemists.

[vms3623-bib-0009] Blentic, A. , Gale, E. , & Maden, M. (2003). Retinoic acid signalling centres in the avian embryo identified by sites of expression of synthesising and catabolising enzymes. Developmental Dynamics: An Official Publication of the American Association of Anatomists, 227(1), 114–127. 12701104 10.1002/dvdy.10292

[vms3623-bib-0010] Böhm, F. , Tinkler, J. H. , & Truscott, T. G. (1995). Carotenoids protect against cell membrane damage by the nitrogen dioxide radical. Nature Medicine, 1(2), 98. 10.1038/nm0295-98 7585018

[vms3623-bib-0011] Boileau, T. W. M. , Boileau, A. C. , & Erdman Jr, J. W. (2002). Bioavailability of all‐trans and cis–Isomers of Lycopene. Experimental Biology and Medicine, 227(10), 914–919. 12424334 10.1177/153537020222701012

[vms3623-bib-0012] Brooks, J. D. , & Thompson, L. U. (2005). Mammalian lignans and genistein decrease the activities of aromatase and 17β‐hydroxysteroid dehydrogenase in MCF‐7 cells. The Journal of Steroid Biochemistry and Molecular Biology, 94(5), 461–467. 15876411 10.1016/j.jsbmb.2005.02.002

[vms3623-bib-0013] Bruggeman, V. , Van As, P. , & Decuypere, E. (2002). Developmental endocrinology of the reproductive axis in the chicken embryo. Comparative Biochemistry and Physiology Part A: Molecular & Integrative Physiology, 131(4), 839–846. 10.1016/s1095-6433(02)00022-3 11897195

[vms3623-bib-0014] Choi, I. H. , Park, W. Y. , & Kim, Y. J. (2010). Effects of dietary garlic powder and α‐tocopherol supplementation on performance, serum cholesterol levels, and meat quality of chicken. Poultry Science, 89(8), 1724–1731. 10.3382/ps.2009-00052 20634529

[vms3623-bib-0015] Correa, S. M. , Adkins‐Regan, E. , & Johnson, P. A. (2005). High progesterone during avian meiosis biases sex ratios toward females. Biology Letters, 1(2), 215–218. 17148170 10.1098/rsbl.2004.0283 PMC1626207

[vms3623-bib-0016] Cunningham, E. J. , & Russell, A. F. (2001). Maternal investment: Sex differences in avian yolk hormone levels. Nature, 412(6846), 498–499. 11484039 10.1038/35087652

[vms3623-bib-0017] Dabrosin, C. , Chen, J. , Wang, L. , & Thompson, L. U. (2002). Flaxseed inhibits metastasis and decreases extracellular vascular endothelial growth factor in human breast cancer xenografts. Cancer Letters, 185(1), 31–37. 12142076 10.1016/s0304-3835(02)00239-2

[vms3623-bib-0018] Dibner, J. J. , & Richards, J. D. (2005). Antibiotic growth promoters in agriculture: History and mode of action. Poultry Science, 84(4), 634–643. 10.1093/ps/84.4.634 15844822

[vms3623-bib-0019] Duffy, T. A. , Picha, M. E. , Won, E. T. , Borski, R. J. , McElroy, A. E. , & Conover, D. O. (2010). Ontogenesis of gonadal aromatase gene expression in atlantic silverside (*Menidia menidia*) Populations with genetic and temperature‐dependent sex determination. Journal of Experimental Zoology Part A: Ecological and Integrative Physiology, 313(7), 421–431. 10.1002/jez.612 20623799

[vms3623-bib-0020] Duncan, D. B. (1955). Multiple range and multiple F‐test. Biometrics, 11, 1–42.

[vms3623-bib-0021] Duncan, K. A. , & Saldanha, C. J. (2011). Neuroinflammation induces glial aromatase expression in the uninjured songbird brain. Journal of Neuroinflammation, 8(1), 81. 21767382 10.1186/1742-2094-8-81 PMC3158750

[vms3623-bib-0022] Edens, F. W. (2011). Gender, age and reproductive status effects on serum prolactin concentrations in different varieties and species of poultry. International Journal of Poultry Science, 10, 832–838.

[vms3623-bib-0023] Edmunds, K. M. , Holloway, A. C. , Crankshaw, D. J. , Agarwal, S. K. , & Foster, W. G. (2005). The effects of dietary phytoestrogens on aromatase activity in human endometrial stromal cells. Reproduction Nutrition Development, 45(6), 709–720. 10.1051/rnd:2005055 16285913

[vms3623-bib-0024] Elbrecht, A. , & Smith, R. G. (1992). Aromatase enzyme activity and sex determination in chickens. Science, 255(5043), 467–470. 1734525 10.1126/science.1734525

[vms3623-bib-0025] Farner, D. S. , King, J. R. , & Parkes, K. C. (Eds.). (2012). Avian biology. Academic Press.

[vms3623-bib-0026] Fazli, N. , Hassanabadi, A. , Mottaghitalab, M. , & Hajati, H. (2015). Manipulation of broiler chickens sex differentiation by *In ovo* injection of aromatase inhibitors, and garlic and tomato extracts. Poultry Science, 94(11), 2778–2783. 10.3382/ps/pev236 26362979

[vms3623-bib-0027] Fisher, C. , & Kemp, C. (2000). Impact of breeder nutrition on broiler performance. International Hatchery Practice, 15, 13–15.

[vms3623-bib-0028] Gao, Y. Y. , Ji, J. , Jin, L. , Sun, B. L. , Xu, L. H. , Wang, C. K. , & Bi, Y. Z. (2016). Xanthophyll supplementation regulates carotenoid and retinoid metabolism in hens and chicks. Poultry Science, 95(3), 541–549. 10.3382/ps/pev335 26574032

[vms3623-bib-0029] Gasnier, C. , Dumont, C. , Benachour, N. , Clair, E. , Chagnon, M. C. , & Séralini, G. E. (2009). Glyphosate‐based herbicides are toxic and endocrine disruptors in human cell lines. Toxicology, 262(3), 184–191. 19539684 10.1016/j.tox.2009.06.006

[vms3623-bib-0030] Gomez‐Prieto, M. S. , Caja, M. M. , & Santa‐Maria, G. (2002). Solubility in supercritical carbon dioxide of the predominant carotenes of tomato skin. Journal of the American Oil Chemists Society, 79(9), 897–902.

[vms3623-bib-0031] Gouranton, E. , Thabuis, C. , Riollet, C. , Malezet‐Desmoulins, C. , El Yazidi, C. , Amiot, M. J. , & Landrier, J. F. (2011). Lycopene inhibits proinflammatory cytokine and chemokine expression in adipose tissue. The Journal of Nutritional Biochemistry, 22(7), 642–648. 20952175 10.1016/j.jnutbio.2010.04.016

[vms3623-bib-0032] Griffin, H. D. , Guo, K. , Windsor, D. , & Butterwith, S. C. (1992). Adipose tissue lipogenesis and fat deposition in leaner broiler chickens. The Journal of Nutrition, 122(2), 363–368. 1732477 10.1093/jn/122.2.363

[vms3623-bib-0033] Guideline, I. H. T. (1994). Text on validation of analytical procedures. International Conference on Harmonization , Geneva.

[vms3623-bib-0034] Hayashi, K. , Nagai, Y. , Ohtsuka, A. , & Tomita, Y. (1994). Effects of dietary corticosterone and trilostane on growth and skeletal muscle protein turnover in broiler cockerels. British Poultry Science, 35(5), 789–798. 10.1080/00071669408417743 7719742

[vms3623-bib-0035] Hutson, J. , Ikawa, H. , & Donahoe, P. K. (1981). The ontogeny of Mullerian inhibiting substance in the gonads of the chicken. Journal of Pediatric Surgery, 16(6), 822–827. 6896069 10.1016/s0022-3468(81)80827-5

[vms3623-bib-0036] Ibrahim, A. R. , & Abul‐Hajj, Y. J. (1990). Aromatase inhibition by flavonoids. The Journal of Steroid Biochemistry and Molecular Biology, 37(2), 257–260. 2268557 10.1016/0960-0760(90)90335-i

[vms3623-bib-0037] Ide, N. , & Lau, B. H. (2001). Garlic compounds minimize intracellular oxidative stress and inhibit nuclear factor‐κB activation. The Journal of Nutrition, 131(3), 1020S–1026S. 11238809 10.1093/jn/131.3.1020S

[vms3623-bib-0038] Itoh, Y. , Replogle, K. , Kim, Y. H. , Wade, J. , Clayton, D. F. , & Arnold, A. P. (2010). Sex bias and dosage compensation in the zebra finch versus chicken genomes: General and specialized patterns among birds. Genome Research, 20(4), 512–518. 20357053 10.1101/gr.102343.109 PMC2847754

[vms3623-bib-0039] Jain, C. K. , Agarwal, S. , & Rao, A. V. (1999). The effect of dietary lycopene on bioavailability, tissue distribution, *in vivo* antioxidant properties and colonic preneoplasia in rats. Nutrition Research, 19(9), 1383–1391.

[vms3623-bib-0040] Jiang, H. , Wang, Z. , Ma, Y. , Qu, Y. , Lu, X. , & Luo, H. (2015). Effects of dietary lycopene supplementation on plasma lipid profile, lipid peroxidation and antioxidant defense system in feedlot Bamei lamb. Asian‐Australasian Journal of Animal Sciences, 28(7), 958. 26104400 10.5713/ajas.14.0887 PMC4478505

[vms3623-bib-0041] Josso, N. , & Picard, J. Y. (1986). Anti‐Mullerian hormone. Physiological Reviews, 66(4), 1038–1090. 3532142 10.1152/physrev.1986.66.4.1038

[vms3623-bib-0042] Josso, N. , di Clemente, N. , & Gouédard, L. (2001). Anti‐Müllerian hormone and its receptors. Molecular and Cellular Endocrinology, 179(1–2), 25–32. 11420127 10.1016/s0303-7207(01)00467-1

[vms3623-bib-0043] Karadas, F. , Surai, P. , Grammenidis, E. , Sparks, N. H. C. , & Acamovic, T. (2006). Supplementation of the maternal diet with tomato powder and marigold extract: Effects on the antioxidant system of the developing quail. British Poultry Science, 47(2), 200–208. 10.1080/00071660600611003 16641031

[vms3623-bib-0044] Koutsos, E. A. , Clifford, A. J. , Calvert, C. C. , & Klasing, K. C. (2003). Maternal carotenoid status modifies the incorporation of dietary carotenoids into immune tissues of growing chickens (*Gallus gallus domesticus*). The Journal of Nutrition, 133(4), 1132–1138. 12672931 10.1093/jn/133.4.1132

[vms3623-bib-0045] Kuroiwa, A. (2018). Sex determination and differentiation in birds. In K. Kobayashi, T. Kitano, Y. Iwao, & M. Kondo (eds), Reproductive and developmental strategies (pp. 391–405). Springer.

[vms3623-bib-0046] Lee, S. N. , Kim, N. S. , & Lee, D. S. (2003). Comparative study of extraction techniques for determination of garlic flavor components by gas chromatography–mass spectrometry. Analytical and Bioanalytical Chemistry, 377(4), 749–756. 12923610 10.1007/s00216-003-2163-z

[vms3623-bib-0047] Li, W. M. , Feng, Y. P. , Zhao, R. X. , Fan, Y. Z. , Affara, N. A. , Wu, J. J. , & Zhang, S. J. (2008). Sex ratio bias in early‐dead embryos of chickens collected during the first week of incubation. Poultry Science, 87(11), 2231–2233. 10.3382/ps.2008-00139 18931172

[vms3623-bib-0048] Lin, M. , Thorne, M. H. , Martin, I. C. , Sheldon, B. L. , & Jones, R. C. (1995). Development of the gonads in the triploid (ZZW and ZZZ) fowl, Gallus domesticus, and comparison with normal diploid males (ZZ) and females (ZW). Reproduction, Fertility and Development, 7(5), 1185–1197. 10.1071/rd9951185 8848586

[vms3623-bib-0049] ‏Luo, C. , & Wu, X. G. (2011). Lycopene enhances antioxidant enzyme activities and immunity function in N‐methyl‐N′‐nitro‐N‐nitrosoguanidine–induced gastric cancer rats. International Journal of Molecular Sciences, 12(5), 3340–3351. 21686188 10.3390/ijms12053340 PMC3116194

[vms3623-bib-0050] Matsushita, S. , Yamashita, J. , Iwasawa, T. , Tomita, T. , & Ikeda, M. (2006). Effects of *in ovo* exposure to imazalil and atrazine on sexual differentiation in chick gonads. Poultry Science, 85(9), 1641–1647. 10.1093/ps/85.9.1641 16977851

[vms3623-bib-0051] Moniot, B. , Declosmenil, F. , Barrionuevo, F. , Scherer, G. , Aritake, K. , Malki, S. , Marzi, L. , Cohen‐Solal, A. , Georg, I. , Klattig, J. , Englert, C. , Kim, Y. , Capel, B. , Eguchi, N. , Urade, Y. , Boizet‐Bonhoure, B. , & Poulat, F. (2009). The PGD2 pathway, independently of FGF9, amplifies SOX9 activity in Sertoli cells during male sexual differentiation. Development, 136(11), 1813–1821. 19429785 10.1242/dev.032631 PMC4075598

[vms3623-bib-0052] Nakabayashi, O. , Kikuchi, H. , Kikuchi, T. , & Mizuno, S. (1998). Differential expression of genes for aromatase and estrogen receptor during the gonadal development in chicken embryos. Journal of Molecular Endocrinology, 20(2), 193–202. 9584834 10.1677/jme.0.0200193

[vms3623-bib-0053] Navara, K. J. (2013). Hormone‐mediated adjustment of sex ratio in vertebrates. Integrative and Comparative Biology, 53(6), 877–887. 23892413 10.1093/icb/ict081

[vms3623-bib-0054] Navara, K. J. , Hill, G. E. , & Mendonça, M. T. (2006). Yolk testosterone stimulates growth and immunity in house finch chicks. Physiological and Biochemical Zoology, 79(3), 550–555. 16691520 10.1086/501054

[vms3623-bib-0055] Nishikimi, H. , Kansaku, N. , Saito, N. , Usami, M. , Ohno, Y. , & Shimada, K. (2000). Sex differentiation and mRNA expression of p 450 c 17, p 450arom and AMH in gonads of the chicken. Molecular Reproduction and Development, 55(1), 20–30. 10602270 10.1002/(SICI)1098-2795(200001)55:1<20::AID-MRD4>3.0.CO;2-E

[vms3623-bib-0056] Oréal, E. , Mazaud, S. , Picard, J. Y. , Magre, S. , & Carré‐Eusèbe, D. (2002). Different patterns of anti‐Müllerian hormone expression, as related to DMRT1, SF‐1, WT1, GATA‐4, Wnt‐4, and Lhx9 expression, in the chick differentiating gonads. Developmental Dynamics, 225(3), 221–232. 12412004 10.1002/dvdy.10153

[vms3623-bib-0057] Orlov, M.V. (1987). Biologicheskii control’ v inkubatsii (biological control in incubation), Moscow (3rd ed.). Russcellezgat.

[vms3623-bib-0058] ‏ Ottinger, M. A. , & vom Saal, F. S. (2002). Impact of environmental endocrine disruptors on sexual differentiation in birds and mammals. Hormones, brain and behavior (pp. 325–383).‏ Academic Press.

[vms3623-bib-0059] Parker, K. L. , & Schimmer, B. P. (1997). Steroidogenic factor 1: A key determinant of endocrine development and function. Endocrine Reviews, 18(3), 361–377. 9183568 10.1210/edrv.18.3.0301

[vms3623-bib-0060] Pelissero, C. , Lenczowski, M. J. P. , Chinzi, D. , Davail‐Cuisset, B. , Sumpter, J. P. , & Fostier, A. (1996). Effects of flavonoids on aromatase activity, an in vitro study. The Journal of Steroid Biochemistry and Molecular Biology, 57(3–4), 215–223. 8645631 10.1016/0960-0760(95)00261-8

[vms3623-bib-0061] Petek, M. , Orman, A. , Dikmen, S. , & Alpay, F. (2008). Relations between day‐old chick length and body weight in broiler, quail and layer. Veteriner Fakültesi Dergisi, Uludağ Üniversitesi, 27(1/2), 25–28.

[vms3623-bib-0062] Pourali, M. , Mirghelenj, S. A. , & Kermanshahi, H. (2010). Effect of garlic powder on productive performance and immune response of broiler chickens challenged with Newcastle disease virus. Global Veterineria, 4(4), 616–621.

[vms3623-bib-0063] Prakash, A. , & Kumar, A. (2013). Lycopene protects against memory impairment and mito‐oxidative damage induced by colchicine in rats: An evidence of nitric oxide signaling. European Journal of Pharmacology, 721(1), 373–381. 24075937 10.1016/j.ejphar.2013.08.016

[vms3623-bib-0064] Puvača, N. , Ljubojević, D. , Kostadinović, L. J. , Lukač, D. , Lević, J. , Popović, S. , & Đuragić, O. (2015). Spices and herbs in broilers nutrition: Effects of garlic (*Allium sativum L*.) on broiler chicken production. World's Poultry Science Journal, 71(3), 533–538.

[vms3623-bib-0065] Raeesi, M. , Hoseini‐Aliabad, S. A. , Roofchaee, A. , Shahneh, A. Z. , & Pirali, S. (2010). Effect of periodically use of garlic (*Allium sativum*) powder on performance and carcass characteristics in broiler chickens. World Academy of Science, Engineering and Technology, 4(68), 1213–1219.

[vms3623-bib-0066] Rao, A. V. , & Agarwal, S. (1999). Role of lycopene as antioxidant carotenoid in the prevention of chronic diseases: A review. Nutrition Research, 19(2), 305–323.

[vms3623-bib-0067] Rissanen T. (2006). Lycopene and cardiovascular disease. In A. V. Rao (Ed.), *Tomatoes, lycopene and human health* (. 141–152). Caledonian Science Press.

[vms3623-bib-0068] Robinzon, B. , Rozenboim, I. , Arnon, E. , & Snapir, N. 1990.The effect of tamoxifen on semen fertilization capacity in White Leghorn male chicks. Poultry Science, 69(7), 1220–1222. 10.3382/ps.0691220 2235838

[vms3623-bib-0069] Romanonoff A.L. (1960). The avian embryo: Structural and functional development. Macmillan Ltd.

[vms3623-bib-0070] Roselli, C. E. , & Klosterman, S. A. (1998). Sexual differentiation of aromatase activity in the rat brain: Effects of perinatal steroid exposure. Endocrinology, 139(7), 3193–3201. 9645693 10.1210/endo.139.7.6101

[vms3623-bib-0071] Rosenstrauch, A. , Degen, A. , Bedrak, E. , & Friedlander, M. (1986). Improvement of fertility in Cornish roosters by the use of clomiphene citrate. Proc. 7th European. Poultry Conference, 1025–1028.

[vms3623-bib-0072] Rubolini, D. , Romano, M. , Martinelli, R. , & Saino, N. (2006). Effects of elevated yolk testosterone levels on survival, growth and immunity of male and female yellow‐legged gull chicks. Behavioral Ecology and Sociobiology, 59(3), 344–352.

[vms3623-bib-0073] Sahin, K. , Yazlak, H. , Orhan, C. , Tuzcu, M. , Akdemir, F. , & Sahin, N. (2014). The effect of lycopene on antioxidant status in rainbow trout (*Oncorhynchus mykiss*) reared under high stocking density. Aquaculture, 418, 132–138.

[vms3623-bib-0074] Sahin, N. , Sahin, K. , Onderci, M. , Karatepe, M. , Smith, M. O. , & Kucuk, O. (2006). Effects of dietary lycopene and vitamin E on egg production, antioxidant status and cholesterol levels in Japanese quail. Asian Australasian Journal of Animal Sciences, 19(2), 224.

[vms3623-bib-0075] SAS . (2012). SAS/TAT user's guide version 9 (1st ed.). SAS Institute Inc.

[vms3623-bib-0076] Sasanami, T. (2017). *Avian* r*eproduction: From* behavior to molecules. (Vol. 1001). Springer.

[vms3623-bib-0077] Sekido, R. , & Lovell‐Badge, R. (2008). Sex determination involves synergistic action of SRY and SF1 on a specific Sox9 enhancer. Nature, 453(7197), 930. 18454134 10.1038/nature06944

[vms3623-bib-0078] Shimada, K. (1998). Gene expression of steroidogenic enzymes in chicken embryonic gonads. Journal of Experimental Zoology Part A: Ecological Genetics and Physiology, 281(5), 450–456. 9662831

[vms3623-bib-0079] Shimada, K. (2002). Sex determination and sex differentiation. Avian and Poultry Biology Reviews, 13(1), 1–14.

[vms3623-bib-0080] Shojai, T. M. , Langeroudi, A. G. , Karimi, V. , Barin, A. , & Sadri, N. (2016). The effect of *Allium sativum* (Garlic) extract on infectious bronchitis virus in specific pathogen free embryonic egg. Avicenna Journal of Phytomedicine, 6(4), 458–467. 27516987 PMC4967842

[vms3623-bib-0081] Skiepko, N. , Chwastowska‐Siwiecka, I. , Kondratowicz, J. , & Mikulski, D. (2016). Fatty acid profile, total cholesterol, vitamin content, and TBARS value of turkey breast muscle cured with the addition of lycopene. Poultry Science, 95(5), 1182–1190. 10.3382/ps/pew005 26908896

[vms3623-bib-0082] Smith, C. A. , Andrews, J. E. , & Sinclair, A. H. (1997). Gonadal sex differentiation in chicken embryos: Expression of estrogen receptor and aromatase genes. The Journal of Steroid Biochemistry and Molecular Biology, 60(5–6), 295–302. 9219920 10.1016/s0960-0760(96)00196-3

[vms3623-bib-0083] Smith, C. A. , McClive, P. J. , Hudson, Q. , & Sinclair, A. H. (2005). Male‐specific cell migration into the developing gonad is a conserved process involving PDGF signalling. Developmental Biology, 284(2), 337–350. 16005453 10.1016/j.ydbio.2005.05.030

[vms3623-bib-0084] Smith, C. A. , McClive, P. J. , Western, P. S. , Reed, K. J. , & Sinclair, A. H. (1999). Evolution: Conservation of a sex‐determining gene. Nature, 402(6762), 601. 10604464 10.1038/45130

[vms3623-bib-0085] Smith, C. A. , Roeszler, K. N. , Bowles, J. , Koopman, P. , & Sinclair, A. H. (2008). Onset of meiosis in the chicken embryo; evidence of a role for retinoic acid. BMC Developmental Biology, 8(1), 85. 18799012 10.1186/1471-213X-8-85 PMC2564928

[vms3623-bib-0086] Stanaćev, V. , Glamočić, D. , Milošević, N. , Perić, L. , Puvača, N. , Stanaćev, V. , Milić, D., & Plavša, N. (2012). Influence of garlic (*Allium sativum L*.) and copper as phytoadditives in the feed on the content of cholesterol in the tissues of the chickens. Journal of Medicinal Plants Research, 6(14), 2816–2819.

[vms3623-bib-0087] Stanaćev, V. , Milošević, N. , Plavša, N. , Bjedov, S. , Stanaćev, V. , Puvača, N., & Arapović , Ž. (2010). Phyto additives (*Allium sativum L*.) in the diet of fattening chickens. Proceedings of the 14th International Symposium of Feed Technology, Novi Sad , 295–302.

[vms3623-bib-0088] Suleria, H. A. R. , Butt, M. S. , Khalid, N. , Sultan, S. , Raza, A. , Aleem, M. , & Abbas, M. (2015). Garlic (*Allium sativum*): Diet‐based therapy of 21^st^ century—A review. Asian Pacific Journal of Tropical Disease, 5(4), 271–278.

[vms3623-bib-0089] Sun, B. , Chen, C. , Wang, W. , Ma, J. , Xie, Q. , Gao, Gao Y. , Chen F. , Zhang X. , & Bi Y. (2015). Effects of lycopene supplementation in both maternal and offspring diets on growth performance, antioxidant capacity and biochemical parameters in chicks. Journal of Animal Physiology and Animal Nutrition, 99(1), 42–49. 24773606 10.1111/jpn.12196

[vms3623-bib-0090] Surai, P. F. , Fisinin, V. I. , & Karadas, F. (2016). Antioxidant systems in chick embryo development. Part 1. Vitamin E, carotenoids and selenium. Animal Nutrition, 2(1), 1–11. 29767100 10.1016/j.aninu.2016.01.001 PMC5941026

[vms3623-bib-0091] Surai, P. F. , Noble, R. C. , & Speake, B. K. (1996). Tissue‐specific differences in antioxidant distribution and susceptibility to lipid peroxidation during development of the chick embryo. Biochimica et Biophysica Acta (BBA)‐Lipids and Lipid Metabolism, 1304(1), 1–10. 8944745 10.1016/s0005-2760(96)00099-9

[vms3623-bib-0092] Takasova, J. , Drdak, M. , & Minarovicova, I. (1995). Characteristics of lipids in tomato seeds. Nahrung, 39(3), 244–245.

[vms3623-bib-0093] T ollba, A. A. H. , & Hassan, M. S. H . (2003). Using some natural additives to improve physiological and productive performance of broiler chicks under high temperature conditions. Black cumin (*Niglla sativa*) or garlic (*Allium sativum*). Egypt Poultry Science, 23(2), 327–340.

[vms3623-bib-0094] Upaganlawar, A. B. , & Balaraman, R. (2012). Cardioprotective effect of vitamin E in combination with lycopene on lipid Profile, lipid metabolizing enzymes and infarction size in myocardial infarction induced by isoproterenol. Pharmacologia, 3(3), 215–220.

[vms3623-bib-0095] Vaillant, S. , Dorizzi, M. , Pieau, C. , & Richard‐Mercier, N. (2001). Sex reversal and aromatase in chicken. Journal of Experimental Zoology Part A: Ecological Genetics and Physiology, 290(7), 727–740. 10.1002/jez.1123 11748621

[vms3623-bib-0096] Valizadeh, E. , & Seratinouri, H. (2013). Effects of garlic extract, anti‐estrogens, and aromatase inhibitor on sex differentiation in embryo. International Journal of Women's Health and Reproduction Sciences, 1(2), 51–55.

[vms3623-bib-0097] Varmaghany, S. , Karimi Torshizi, M. A. , Rahimi, S. , Lotfollahian, H. , & Hassanzadeh, M. (2015). The effects of increasing levels of dietary garlic bulb on growth performance, systolic blood pressure, hematology, and ascites syndrome in broiler chickens. Poultry Science, 94(8), 1812–1820. 10.3382/ps/pev148 26049796

[vms3623-bib-0098] Wang, C. , Mäkelä, T. , Hase, T. , Adlercreutz, H. , & Kurzer, M. S. (1994). Lignans and flavonoids inhibit aromatase enzyme in human preadipocytes. The Journal of Steroid Biochemistry and Molecular Biology, 50(3–4), 205–212. 8049151 10.1016/0960-0760(94)90030-2

[vms3623-bib-0099] Wibbels, T. , Bull, J. J. , & Crews, D. (1992). Steroid hormone‐induced male sex determination in an amniotic vertebrate. Journal of Experimental Zoology Part A: Ecological Genetics and Physiology, 262(4), 454–457. 10.1002/jez.1402620413 1624917

[vms3623-bib-0100] Yamamoto, I. , Tsukada, A. , Saito, N. , & Shimada, K. (2003). Profiles of mRNA expression of genes related to sex differentiation of the gonads in the chicken embryo. Poultry Science, 82(9), 1462–1467. 10.1093/ps/82.9.1462 12967261

[vms3623-bib-0101] Yoshida, K. , Shimada, K. , & Saito, N. (1996). Expression of P45017 α‐hydroxylase and P450 Aromatase genes in the chicken gonad before and after sexual differentiation. General and Comparative Endocrinology, 102(2), 233–240. 8998967 10.1006/gcen.1996.0064

[vms3623-bib-0102] Zhang, C. , Saito, N. , Matsuda, Y. , & Shimada, K. (1998). Identification of sperm‐bearing female‐specific chromosome in the sex‐reversed chicken. Journal of Experimental Zoology, 280(1), 65–72. 10.1002/(sici)1097-010x(19980101)280:1<65::aid-jez8>3.0.co;2-f 9437853

